# A Cross-Sectional Assessment of Urinary Tract Infections Among Geriatric Patients: Prevalence, Medication Regimen Complexity, and Factors Associated With Treatment Outcomes

**DOI:** 10.3389/fpubh.2021.657199

**Published:** 2021-10-18

**Authors:** Ali Akhtar, Mohamed Azmi Ahmad Hassali, Hadzliana Zainal, Irfhan Ali, Amer Hayat Khan

**Affiliations:** ^1^Discipline of Clinical Pharmacy, School of Pharmaceutical Sciences, Universiti Sains Malaysia, George Town, Malaysia; ^2^Discipline of Social and Administrative Pharmacy, School of Pharmaceutical Sciences, Universiti Sains Malaysia, George Town, Malaysia; ^3^Hospital Pulau Pinang, Ministry of Health, George Town, Malaysia

**Keywords:** asymptomatic bacteriuria, cystitis, medication complexity, outcomes, urinary tract infections

## Abstract

**Background:** Urinary tract infections (UTIs) are the second most prevalent infection among the elderly population. Hence, the current study aimed to evaluate the prevalence of UTIs among older adults, medication regimen complexity, and the factors associated with the treatment outcomes of elderly patients infected with UTIs.

**Methods:** A retrospective cross-sectional study was conducted at the Department of Urology, Hospital Pulau Pinang, Malaysia. The patients ≥65 years of age were included in the present study with a confirmed diagnosis of UTIs from 2014 to 2018 (5 years).

**Results:** A total of 460 patients met the inclusion criteria and were included in the present study. Cystitis (37.6%) was the most prevalent UTI among the study population followed by asymptomatic bacteriuria (ASB) (31.9%), pyelonephritis (13.9%), urosepsis (10.2%), and prostatitis (6.4%). Unasyn (ampicillin and sulbactam) was used to treat the UTIs followed by Bactrim (trimethoprim/sulfamethoxazole), and ciprofloxacin. The factors associated with the treatment outcomes of UTIs were gender (odd ratio [*OR*] = 1.628; *p* = 0.018), polypharmacy (*OR* = 0.647; *p* = 0.033), and presence of other comorbidities (*OR* = 2.004; *p* = 0.002) among the study population.

**Conclusion:** Cystitis is the most common UTI observed in older adults. Gender, the burden of polypharmacy, and the presence of comorbidities are the factors that directly affect the treatment outcomes of UTIs among the study population.

## Introduction

Urinary tract infections (UTIs) are the most common type of infection among the elderly population around the world and the most common cause of hospitalization due to bacterial infections (1). Generally, a UTI is defined as an infection in the urinary tract system which may include both upper urinary and lower urinary tracts (2). Approximately 7 million hospital visits, 1 million emergency visits, and 100,000 hospitalizations are due to UTIs which are around 25% of all infections among older people every year (3). Approximately, the overall incidence of UTIs among older men and women ranges in one infection per 14–20 persons-years (4). The treatment and diagnosis of UTIs are more difficult among the elderly population as compared with the younger individuals because of many underlying risk factors, such as older age, spinal cord injuries, diabetes mellitus, impaired immune conditions, and most importantly catheterization (4).

Older adults are more prone to UTIs as compared with young individuals due to the high rates of urinary retention, urinary incontinence, long-term hospitalizations, presence of comorbidities, accompanying urinary catheterizations, and declining immune responses (5, 6). Modifiable risk factors of UTIs among older people include urinary tract abnormalities particularly in those with urinary retention or incontinence (e.g., prostatic hyperplasia), diabetes mellitus, urinary catheterization, and sexual intercourse, which is the major risk factor for both men and women in older age (7).

The prevalence of UTI is higher in women as compared with men in all age groups. In sexually active young women, the incidence of UTI ranging from 0.5 to 0.7 per person-year (8), however, in young men it is 0.01 per person-year. In middle age, the incidence of UTI decreases but it increases with the increase in age (2). An estimate of 10% of women aged more than 65 years reported UTI in the last 12 months (9), whereas this number increase up to 30% in women aged more than 85 years (10). In a study on postmenopausal women, the incidence of UTI has been reported as 0.07 per person-year and 0.12 per person-year in women with uncontrolled diabetes mellitus (4). The incidence of UTI significantly increases in both men and women aged more than 85 years.

Polypharmacy is the major risk factor for overactive bladder syndrome in older adults (11). “Overactive bladder syndrome” is the complex of symptoms that include the sudden need to urinate with the fear of involuntary leakage, nocturia, leakage of urine prior to urine intention, and pollakiuria. Some drugs stimulate incontinence as their adverse effect and some of the drugs have interactions between them that increase the chances of overactive bladder syndrome (11).

The clinical presentation of UTIs among older adults leads to complexity in the diagnosis due to localized urinary symptoms and typical clinical history as compared with the young individuals. The increased prevalence of asymptomatic bacteriuria (ASB) among the elderly population may lead to more difficulty in the diagnosis of UTIs (1U). In primary and secondary care, empirical antibiotics are prescribed for a suspected UTI in which more than 50% of the prescribed antibiotics are considered unnecessary among elderly patients (12). To reduce the threat of antibiotic resistance to public health, antibiotic stewardship programs and national guidelines for the rational use of antibiotics have been adapted to control this situation (13).

In the current study, we evaluated the prevalence of different UTIs, medication regimen complexity, and identify different risk factors involved in the treatment outcomes of UTIs among geriatric patients.

## Methods

### Design of Study, Setting, and Time

We conducted a retrospective cross-sectional study at the urology department of a tertiary care public hospital (Hospital Pulau Pinang) in the northern territory of Malaysia. Data were collected from the record room of the urology department from October 2019 to February 2020.

### Study Population

We evaluated all the medical records of older adults from January 2014 to December 2018 (5 years) by using a convenience sampling technique. The inclusion criteria of the participants were: patients aged ≥ 65 years, complete medical and clinical information in the record, should have a UTI episode confirmed by the physician. Patients <65 years of age, incomplete records, and no information on UTI episodes were excluded from the study.

### Ethical Approval and Data Collection

**This** study was approved by the National Institute of Health and the Medical Research and Ethics Committee, Malaysia (NMRR-19-1037-46721) prior to starting the data collection. The **s**ocio-demographic and therapeutic data of the included participants were collected with the help of a comprehensive data collection form from the record room of the urology department, Hospital Pulau Pinang, Malaysia. All the parameters of the data collection form are presented in Table 1. The records of prescribed medicines of the included participants were also collected to assess the medication complexity by using the medication regimen complexity index (MRCI) (14).

**Table 1:** Sociodemographic characteristics of the study population.

**Characteristics**	***N* (%)**	**Treatment outcomes**		***p*-value**
		**Improved**	**Not improved**	
**Gender**				0.017*
Male	181 (39.3)	114 (24.8)	67 (14.6)	
Female	279 (60.7)	205 (44.6)	74 (16.1)	
**Age (years)**				
65–75	342 (74.3)	246 (53.5)	96 (20.9)	0.041*
>75	118 (25.7)	73 (15.9)	45 (9.8)	
**Marital status**				
Single	22 (4.8)	12 (2.6)	10 (2.2)	
Married	256 (55.7)	186 (40.4)	70 (15.2)	0.081
Divorced	47 (10.2)	27 (5.9)	20 (4.3)	
Widow	135 (29.3)	94 (20.4)	41 (8.9)	
**Race**				
Malay	127 (27.6)	93 (20.2)	34 (7.4)	
Chinese	271 (58.9)	185 (40.2)	86 (18.7)	0.509
Indian	62 (13.5)	41 (8.9)	21 (4.6)	
**Home**				
Own Home	428 (93.0)	296 (64.3)	132 (28.7)	0.748
Nursing home	32 (7.0)	23 (5.0)	9 (2.0)	
**Smoking**				
Smoker	148 (32.2)	107 (23.3)	41 (8.9)	0.345
Non-smoker	312 (67.8)	212 (46.1)	100 (21.7)	
**Alcohol**				
Alcoholic	142 (30.9)	95 (20.7)	47 (10.2)	0.447
Non-alcoholic	318 (69.1)	224 (48.7)	94 (20.4)	
**Polypharmacy (number of medications)**				
≤ 5	188 (40.9)	120 (26.1)	68 (14.8)	0.033*
>5	272 (59.1)	199 (43.3)	73 (15.9)	
**Co-morbidities**				
Yes	336 (73.0)	247 (53.7)	89 (19.3)	<0.001*
No	124 (27.0)	72 (15.7)	52 (11.3)	

*Using chi-square. p <0.05.

The MRCI scores were calculated based on the medications prescribed to the patients. It has three sections with 65 items, such as “types of dosage forms,” “frequencies of prescribed medications,” and “other additional instructions regarding medicines.” This tool provides the limitless entry of medicines to a single individual. There is no maximum score of this index, it increases with the increase in the number of medicines, however, the minimum score is 1.5, which indicates that one tablet or capsule once daily.

### Statistical Analysis

**S**tatistical analysis was performed by using SPSS version 24 (SPSS Inc., Chicago, IL, USA). The treatment outcomes of UTIs were compared with the categorical variables using the chi-square test and the *p*-value was considered as significant at < 0.05. The continuous variables were reported as frequencies (percentages). Binary and multiple logistic regression were used to evaluate the association between each independent variable and the treatment outcomes with 95% *CI*, adjusted odds ratio (*OR*), and *p*-values (*p* < 0.05). The MRCI scores were analyzed as continuous variables.

## Results

### Characteristics of the Study Population

In the current 5 year retrospective cross-sectional study, 460 participants were included in which women 279 (60.7%) were in majority as compared with men 181 (39.3%) with a mean age of 72 ± 4 years. Most of the study participants 342 (74.3%) were in the age range of 65–75 years of age and 118 (25.7%) were above 75 years. Majority of the included population was married 256 (55.7%), Chinese 271 (58.9%), non-smokers 312 (67.8%), and non-alcoholic 318 (69.1%). Detailed association between the sociodemographic variables with the treatment outcome parameters (improved or not improved) among the study population infected with UTIs is presented in Table 1.

### Prevalence of UTIs Among the Study Population

The most common UTIs reported in elderly population are cystitis 173 (37.6%) followed by ASB 147 (31.9%), pyelonephritis 64 (13.9%), urosepsis 47 (10.2%), and prostatitis 29 (6.4%). Other co-morbidities present with these UTIs among the study population are diabetes mellitus, hypertension, dyslipidemia, ischemic heart disease, and chronic kidney disease as described in Table 2.

**Table 2:** List of urinary tract infections (UTIs) and other co-morbidities among the study population.

**Urinary tract infections**	***N* (%)**	**Co-morbidities**	***N* (%)**
Cystitis	173 (37.6)	Diabetes mellitus	198 (43.1)
Asymptomatic bacteriuria	147 (31.9)	Hypertension	156 (33.9)
Pyelonephritis	64 (13.9)	Dyslipidaemia	113 (24.5)
Urosepsis	47 (10.2)	Ischemic heart disease	59 (12.8)
Prostatitis	29 (6.4)	Chronic kidney disease	48 (10.4)

Unasyn (ampicillin and sulbactam), Bactrim (trimethoprim/sulfamethoxazole), ciprofloxacin are the most commonly used antibiotics used to treat UTIs in the study population (Table 3).

**Table 3:** List of oral medications among the study population.

**Oral medications**	***N* (%)**
Unasyn (Ampicillin and Sulbactam)	263 (57.1)
Bactrim (Trimethoprim/sulfamethoxazole)	143 (31)
Ciprofloxacin	25 (5.4)
Levofloxacin	17 (3.7)
Cloxacillin	12 (2.6)
Hytrin (Terazosin)	390 (84.8)

### Medication Regimen Complexity

The number of prescribed medications ranges from 1 to 20 with a mean value of 5 medicines per patient included in the current study. Over 59.1% of the included participants are taking more than five medicines simultaneously which leads to the high burden of polypharmacy. The total MRCI score for the study population ranging from 5 to 27 per patient with a median of 14. The MRCI scores in detail are presented in Table 4.

**Table 4:** Medication regimen complexity index by sections.

**MRCI total score**	**Mean (SD)**	**Minimum**	**Maximum**
MRCI section A score	5.85 (2.937)	1	20
MRCI section B score	7.47 (2.890)	2	17
MRCI section C score	0.74 (0.802)	0	4
MRCI total score	14.04 (4.146)	5	27

*Section A: Number of medications prescribed; Section B: Frequency of prescribed medicines; Section C: Additional instructions of prescribed medicines*.

### Factors Associated With the Treatment Outcomes of UTIs Among the Elderly Population

**The** different associated factors involved in the treatment outcomes of UTIs among the elderly population have been predicted by using binary logistic regression analysis. Gender, marital status, age, race, smoking status, alcohol consumption, home, polypharmacy, and presence of co-morbidities are the factors that are analyzed to predict their association with the treatment outcomes of UTIs among the study population. Out of these nine independent variables, only four (gender [*OR* = 1.628; *p* = 0.018], age [*OR* = 1.580; *p* = 0.042], polypharmacy [*OR* = 0.647; *p* = 0.033], and the presence of co-morbidities [OR = 2.004; *p* = 0.002]) of them show statistically significant association with the treatment outcomes in binary logistic regression. These associated variables are then tested in multiple logistic regression, all of them show significant association except the age factor (*OR* = 1.378; *p* = 0.168). Table 5 shows the detailed presentation of binary and multiple logistic regression analysis.

**Table 5:** Predictors affecting the treatment outcomes of UTIs among the study population.

		**Binary logistic regression**				**Multiple logistic regression**			
				**95% CI**				**95% CI**	
**Variables**	***N* (%)**	**Odd ratio**	***p*-value**	**Lower**	**Upper**	**Odd ratio**	***p*-value**	**Lower**	**Upper**
**Gender**									
Male	181 (39.3)	Reference				Reference			
Female	279 (60.7)	1.628	0.018*	1.089	2.434	1.529	0.044*	1.011	2.312
**Age (years)**									
65–75	342 (74.3)	Reference				Reference			
>75	118 (25.7)	1.580	0.042*	1.017	2.453	1.378	0.168	0.873	2.175
**Marital status**									
Single	22 (4.8)	Reference							
Married	256 (55.7)	0.452	0.078	0.187	1.092				
Divorced	47 (10.2)	0.889	0.821	0.321	2.463				
Widow	135 (29.3)	0.523	0.166	0.209	1.308				
**Race**									
Malay	127 (27.6)	Reference							
Chinese	271 (58.9)	1.272	0.315	0.796	2.032				
Indian	62 (13.5)	1.401	0.314	0.727	2.701				
**Home**									
Own Home	428 (93.0)	Reference							
Old Care Home	32 (7.0)	0.877	0.748	0.395	1.948				
**Smoking**									
Smoker	148 (32.2)	Reference							
Non-smoker	312 (67.8)	1.231	0.345	0.800	1.895				
**Alcohol**									
Alcoholic	142 (30.9)	Reference							
Non-alcoholic	318 (69.1)	0.848	0.447	0.555	1.297				
**Polypharmacy**									
≤ 5	188 (40.9)	Reference				Reference			
>5	272 (59.1)	0.647	0.033*	0.434	0.966	0.642	0.033*	0.426	0.966
**Co-morbidities**									
Yes	336 (73.0)	Reference				Reference			
No	124 (27.0)	2.004	0.002*	1.302	3.085	1.872	0.005*	1.205	2.907

*p < 0.05.

## Discussion

Urinary tract infections are the most common type of infections in all age groups, particularly, in older adults due to their compromised immune response and sedentary lifestyle. A UTI can be defined as the presence of a significant number of bacteria (quantitative method) in the urinary tract system which may lead to symptomatic or asymptomatic infection (15).

The current study shows a high prevalence of cystitis (37.6%) among the study population followed by ASB (31.9%), pyelonephritis (13.9%), urosepsis (10.2%), and prostatitis (6.4%). Urine is stored in the bladder and uropathogens can enter and colonize in bladder much easier than other parts of the urinary tract system and cause cystitis (16). Previous literature also reported high prevalence of cystitis among the elderly population than any other type of UTI (17, 18).

Urinary tract infections are more common bacterial infections in women as compared to men of all ages and increases with the increase in age. During the reproductive years, all women have at least one episode of UTI in their life and it increases up to 60% in their postmenopausal years. Hormonal changes, anomalies in the urinary tract, compromised immune system, urinary incontinence, functional disability, nutrition deficiency, and presence of other illnesses are the main risk factors contributing to UTIs in the elderly population (19). Moreover, loss of estrogen in elderly women changes the flora of the vagina, a decrease in the number of lactobacilli in vaginal flora leads to periurethral colonization. Urine production is increased while the capacity of the bladder decreased, decreases in voided volume, and decreases in the urinary tract threshold which ultimately leads to a higher risk of UTIs among older women. The present study also shows the high prevalence of UTIs in women (60.7%) as compared with men (39.3%). A high prevalence of UTIs in women (62.5%) than in men (37.5%) was found by Chaudhary et al. (20). One more study reported a 51.3% prevalence of UTIs in women and 48.6% in men (21).

The choice of antibiotics to treat UTIs among older adults is more complex as compared with young individuals due to the presence of a large range of pathogens, possibility of antibiotic resistance is higher, particularly, in the hospitalized patients or who receive more courses of antibiotics in their life span (22). In the present study, Unasyn (ampicillin and sulbactam) (57.1%) is the most prescribed antibiotic for the treatment of UTIs among the elderly study population followed by Bactrim (trimethoprim/sulfamethoxazole) (31%) and ciprofloxacin (5.4%). Unnecessary antibiotics should not be prescribed to older adults to reduce the risk of mortality and morbidity, moreover, narrow-spectrum antibiotics should be used to treat UTIs among the elderly population. For suspected UTI in the elderly population, the best practice is to send the urine sample for culture and sensitivity and wait for the results rather than to start broad-spectrum empirical therapy to reduce the risk of unnecessary antibiotic use in clinically well patients. If empirically therapy is required, the patterns of previous isolates of sensitivity, resistance patterns, and previous medication records should be evaluated, and the choice of antibiotic should be reassessed after 48–72 h based on the results of urine culture and sensitivity tests (22).

In the polypharmacy of elderly patients, inappropriate or unnecessary prescribing and adverse drug reactions are very common (23). In the present study, polypharmacy (*OR* = 0.642; *p* = 0.033) is one of the most important risk factors involved in the treatment outcomes of UTIs among the elderly population. The majority of the included participants (59.1%) are taking more than five medicines simultaneously for the treatment of their diseases which leads to the high burden of polypharmacy and medication regimen complexity. Older adults taking 5–8 drugs simultaneously were at high risk of hospitalization due to adverse drug reactions as compared with those who were taking 0–4 drugs (23). The clinicians are required to make modifications in the regimen of elderly patients experiencing UTIs to reduce the risk of drug-drug interactions and adverse drug reactions. Women receiving alpha-blockers for their hypertension causes incontinence reported in a case-control study, however, when these antihypertensives were discontinued, almost complete resolution was observed in their urinary symptoms. The use of loop diuretics instead of thiazide among 172 elderly patients with hypertension and heart failure was associated with increased frequency of urine and relaxation of the bladder to reduce the risk of UTIs (24). Previous literature reported cough-induced incontinence after the initiation of ACE inhibitors among older adults and remits after discontinuation (25, 26).

The presence of comorbidities is a significant predictor among the elderly patients with UTIs effecting their treatment outcomes in the present study (*OR* = 1.872; *p* = 0.005). Diabetes mellitus (43.1%) and hypertension (33.9%) are the most common comorbidities present among the study population. Diabetes mellitus effects the immune system of older adults due to autonomic neuropathy that leads to incomplete emptying of the bladder and poor metabolic control, which all contribute to the increased risk of UTIs in elderly patients with diabetes mellitus (27). A similar prevalence of UTIs among diabetic individuals was reported by Pargavi et al. (37%) (28), Yadav et al. (38%) (29), and Sewify et al. (35%) (30).

Our current study has several limitations. First, given the retrospective study design, it is possible that there are confounding factors associated with the treatment decisions and clinical outcomes we did not include. Second, this is a single-center study and may not be generalizable to other settings. Additionally, given the retrospective nature of our study, we were unable to collect physical exam data on infection severity among geriatric patients. Additionally, we excluded a significant number of patients with incomplete medical records, which may limit the impact of our findings. Finally, we did not collect information on antibiotic-associated adverse events, adherence to guidelines by the physicians due to limited data available in the medical records.

## Conclusion

In conclusion, a high prevalence of cystitis (37.6%), asymptomatic bacteriuria (31.9%), and pyelonephritis (13.9%) were observed in the study population of elderly patients. Gender (*OR* = 1.529; *p* = 0.044), polypharmacy (*OR* = 0.642; *p* = 0.033), and presence of other comorbidities (*OR* = 1.872; *p* = 0.005) were the potential risk factors for the treatment outcomes of UTIs in older adults. By reducing the burden of polypharmacy and medication regimen complexity, the treatment outcomes of UTIs among the elderly population could be improved.

## Data Availability Statement

The raw data supporting the conclusions of this article will be made available by the authors, without undue reservation.

## Ethics Statement

The studies involving human participants were reviewed and approved by National Institute of Health and Medical Research and Ethics Committee, Malaysia (NMRR-19-1037-46721). Written informed consent for participation was not required for this study in accordance with the national legislation and the institutional requirements.

## Author Contributions

AA conceptualized and designed the study, conducted the statistical analyses, interpreted the data, and drafted the manuscript. AK, HZ, and MA revised the manuscript for intellectual content, read, and approved the final version of the manuscript. IA helped in data collection and supervised the drafting of the manuscript, supported in interpreting the data, and revised the manuscript for intellectual content. All authors read and approved the final manuscript.

## Conflict of Interest

The authors declare that the research was conducted in the absence of any commercial or financial relationships that could be construed as a potential conflict of interest.

## Publisher's Note

All claims expressed in this article are solely those of the authors and do not necessarily represent those of their affiliated organizations, or those of the publisher, the editors and the reviewers. Any product that may be evaluated in this article, or claim that may be made by its manufacturer, is not guaranteed or endorsed by the publisher.
